# Gastrointestinal Symptoms in Systemic Sclerosis: Prevalence and Severity across Disease Subtypes and Autoantibody Profiles

**DOI:** 10.15388/Amed.2026.33.1.13

**Published:** 2026-07-22

**Authors:** Marina Vujovic Sestakov, Sretko Lukovic, Ivana Pavlovic, Maja Zlatanovic, Predrag Ostojic

**Affiliations:** 1 Institute of Rheumatology, Belgrade, Serbia; 2 Faculty of Medicine, University of Belgrade, Belgrade, Serbia

**Keywords:** systemic sclerosis, gastrointestinal symptoms, patient-reported outcomes, UCLA SCTC GIT 2.0, sisteminė sklerozė, virškinimo trakto simptomai, pacientų nurodyti rezultatai

## Abstract

**Background:**

This study aimed to evaluate the prevalence and severity of gastrointestinal (GI) symptoms in patients with systemic sclerosis (SSc), their impact on social functioning and emotional well-being, and to compare findings between patients with limited cutaneous systemic sclerosis (lcSSc) and diffuse cutaneous systemic sclerosis (dcSSc), as well as between patients positive for anticentromere (ACA) or anti-topoisomerase I (ATA) antibodies.

**Materials and methods:**

Forty-two consecutive patients with SSc were included: 31 with lcSSc and 11 with dcSSc. ACA antibodies were detected in 26 patients, and ATA antibodies in 16 patients. The UCLA SCTC GIT 2.0 self-assessment questionnaire was used to assess the severity of GI symptoms across seven subscales: reflux, distention/bloating, diarrhea, fecal soilage, constipation, social functioning, and emotional well-being.

**Results:**

Symptoms of reflux (lcSSc/dcSSc = 64.5%/81.8%), distention/bloating (lcSSc/dcSSc = 61.3%/81.8%), constipation (lcSSc/dcSSc = 45.2%/45.5%), and fecal soilage (lcSSc/dcSSc = 6.4%/18.2%) were similarly frequent in lcSSc and dcSSc. Diarrhea was more common in dcSSc (45.5% vs. 16.1%, *p* = 0.04), with a higher median index value (0.25 vs. 0.00, *p* = 0.02), thus indicating more severe symptoms. No differences were observed in the prevalence or severity of GI symptoms between ACA- and ATA-positive patients. Median index values for social functioning and emotional well-being did not differ by the disease subtype or antibody status.

**Conclusions:**

Diarrhea was more prevalent and more severe in patients with dcSSc, while other gastrointestinal manifestations showed no major differences between subtypes. Symptom prevalence and severity did not differ according to the antibody status, and their impact on social and emotional functioning was similar across the groups.

## Abstract (it)

**Įvadas:**

Šio tyrimo tikslas – įvertinti virškinimo trakto (VT) simptomų paplitimą ir sunkumą pacientams, sergantiems sistemine skleroze (SSc), jų poveikį socialiniam funkcionavimui ir emocinei gerovei, taip pat palyginti pacientų, sergančių ribota odine sistemine skleroze (lcSSc) ir difuzine odine sistemine skleroze (dcSSc), bei pacientų, kurių kraujyje nustatyta anticentromerinių (ACA) arba antitopoizomerazės I (ATA) antikūnų, rezultatus.

**Medžiaga ir metodai:**

Į tyrimą buvo įtraukti 42 iš eilės atrinkti SSc sergantys pacientai: 31 pacientas su lcSSc ir 11 pacientų su dcSSc. ACA antikūnų buvo aptikta 26 pacientams, o ATA antikūnų – 16 pacientų. UCLA SCTC GIT 2.0 savęs vertinimo klausimynas buvo naudojamas virškinimo trakto simptomų sunkumui įvertinti pagal septynias skalės dalis: refliuksą, pilvo pūtimą, vidurių pūtimą, išmatų nelaikymą, vidurių užkietėjimą, socialinį funkcionavimą ir emocinę savijautą.

**Rezultatai:**

refliukso simptomai (lcSSc/dcSSc = 64,5 %/81,8 %), pilvo pūtimas / išsipūtimas (lcSSc/dcSSc = 61,3 %/81,8 %), vidurių užkietėjimo (lcSSc/dcSSc = 45,2 %/45,5 %) ir išmatų nelaikymo (lcSSc/dcSSc = 6,4 %/18,2 %) simptomai buvo panašiai dažni lcSSc ir dcSSc atvejais. Viduriavimas buvo dažnesnis dcSSc atveju (45,5 %, palyginti su 16,1 %, p = 0,04), o jo indekso mediana buvo didesnė (0,25 su 0,00, p = 0,02), o tai rodo sunkesnius simptomus. ACA ir ATA teigiamų pacientų virškinimo trakto simptomų paplitimas ar sunkumas nesiskyrė. Medianinės socialinio funkcionavimo ir emocinės gerovės indekso vertės nesiskyrė pagal ligos potipį ar antikūnų statusą.

**Išvados:**

Viduriavimas buvo dažnesnis ir sunkesnis pacientų, sergančių dcSSc, o kiti virškinimo trakto simptomai nesiskyrė tarp potipių. Simptomų paplitimas ir sunkumas nesiskyrė pagal antikūnų statusą, o jų poveikis socialiniam ir emociniam funkcionavimui buvo panašus visose grupėse.

## Introduction

Systemic sclerosis (SSc) is a chronic systemic disease characterized by damage to the small blood vessels of the skin and internal organs, activation of the immune system that contributes to vascular and tissue damage, and simultaneous promotion of excessive production of extracellular connective fibers with their deposition in the walls of blood vessels, skin, and internal organs. The deposition of these fibers manifests as the most noticeable clinical feature of the disease – hard and thickened skin (scleroderma), whereas the severity of the disease depends on the degree of involvement of vital visceral organs, primarily the lungs, kidneys, heart, and the digestive system. There are two forms of SSc, depending on the extent of sclerodermatous changes: limited (lcSSc) and diffuse (dcSSc) forms of the disease [1]. After the skin, the gastrointestinal (GI) tract is the most commonly affected organ system in SSc, often representing one of the earliest manifestations of the disease and being involved in around 90% of patients, significantly contributing to a reduced quality of life [2,3]. Any part of the GI tract, from the mouth to the anus, can be affected, leading to variability in symptoms [4,5]. The pathogenesis involves fibrosis and damage to smooth muscle cells, and nerve fibers. In the early stage, there is a disturbance in cholinergic innervation without pronounced morphological changes, whereas, in later stages, there is atrophy of smooth muscle cells and fibrosis of the muscular layer, submucosa, and lamina propria, accompanied by mucosal erosions [6].

Esophageal involvement is the most common gastrointestinal manifestation of SSc, affecting up to 90% of patients and often representing one of the earliest disease features. Dysfunction of the lower esophageal sphincter and smooth muscle weakness in the distal two-thirds of the esophagus lead to gastroesophageal reflux disease and impaired esophageal peristalsis, resulting in symptoms such as heartburn, regurgitation, and dysphagia [7]. Gastric involvement in SSc is common and may manifest as gastroparesis, leading to symptoms such as early satiety, postprandial bloating, nausea, and regurgitation [8]. Small intestinal involvement occurs in a substantial proportion of SSc patients and is characterized by dysmotility and small intestinal bacterial overgrowth, presenting with abdominal distension, bloating, diarrhea, and features of malabsorption [9]. Colonic and anorectal involvement is frequent in SSc and typically presents with hypomotility-related constipation and fecal incontinence, significantly contributing to gastrointestinal morbidity and an impaired quality of life [10]. Given the frequency and clinical impact of GI involvement in SSc, systematic assessment of GI symptoms and their variation according to the disease subtype and autoantibody profile is essential, as effective management remains challenging, and the symptom burden can significantly affect the patients’ quality of life. This study aimed to evaluate the prevalence and severity of GI symptoms in patients with SSc, their impact on social functioning and emotional well-being, and to compare results between patients with lcSSc and dcSSc, as well as those with anticentromere (ACA) and anti-topoisomerase I (ATA) antibodies.

## Materials and Methods

This cross-sectional, single-center study included 42 consecutive patients with systemic sclerosis (SSc) who were followed at a tertiary referral center during 2021. All patients fulfilled the 2013 ACR/EULAR classification criteria for SSc [11], and the study was approved by the local Ethics Committee. Thirty-one patients had lcSSc, while eleven had dcSSc. Exclusion criteria were a previously diagnosed gastrointestinal disease, or any other associated systemic connective tissue disease. In order to assess the GI symptoms and the health-related quality of life (HRQOL), the patient-reported outcome measure the UCLA SCTC GIT 2.0 (University of California, Los Angeles Scleroderma Clinical Trial Consortium Gastrointestinal Tract Instrument) was used. This questionnaire is designed to evaluate the severity of GI symptoms and their impact on social functioning and emotional well-being [12]. The UCLA SCTC GIT 2.0 questionnaire was translated and validated in Serbian [13]. It consists of 34 questions divided into seven subscales. Five subscales address the most common GI symptoms in SSc: reflux, distention/bloating, diarrhea, fecal soilage, and constipation. The remaining two subscales assess the impact of GI symptoms on social functioning and emotional well-being. Responses to individual questions are scored on a scale from 0 (no symptoms) to 3 (severe symptoms), except for diarrhea and constipation, which are scored from 0 to 2 and 0 to 2.5, respectively. The total GI score is calculated as the mean of six out of seven subscales (excluding constipation) and ranges from 0 (better HRQOL) to 2.83 (worse HRQOL) [14].

## Statistical Analyses

All statistical analyses were performed using *IBM SPSS Statistics* version 20.0. Depending on the data distribution, differences between two independent samples were assessed by using either the Student’s *t-*test or the Mann–Whitney *U* test. The chi-square test or Fisher’s exact test was applied to evaluate differences in categorical variable frequencies. Continuous variables are presented as the median and interquartile range (Q1–Q3), and categorical variables are presented as counts (n) and percentages (%). Statistical significance was defined as *p* < 0.05.

## Results

Of the 42 patients enrolled in this study, 39 (92.2%) were women and 3 (7.1%) were men. The mean age was 59.1 ± 9.7 years, and the mean disease duration was 10.0 ± 7.8 years. Thirty-one patients had lcSSc (73.8%), and 11 had dcSSc (26.2%). ACA autoantibodies were detected in 26 patients (61.9%), and ATA autoantibodies in 16 patients (38.1%). Demographic and clinical characteristics of the patients are displayed in Table 1.

**Table 1:** Demographics and clinical characteristics of the study population

Number of patients		42
Sex	female	39 (92.2%)
	male	3 (7.1%)
Age (years), mean ± SD		59.1 ± 9.7
Disease duration (years), mean ± SD		10.0 ± 7.8
Type of disease	limited	31 (73.8%)
	diffuse	11 (26.2%)
Autoantibodies	ACA	26 (61.9%)
	ATA	16 (38.1%)

*Note*. Abbreviations in use: ACA – anticentromere, ATA – anti-topoisomerase-I.

Of 42 patients, 35 (83.3%) reported the presence of GI symptoms. There was no difference in the percentage of patients using proton pump inhibitors between lcSSc and dcSSc (32.3% vs. 27.3%), or between ACA- and ATA-positive patients (34.6% vs. 25%).

In patients with lcSSc, the most commonly reported symptoms were reflux (20/31, 64.5%), followed by bloating (19/31, 61.3%), constipation (14/31, 45.2%), diarrhea (5/31, 16.1%), and, least frequently, fecal soilage due to fecal incontinence (2/31, 6.4%). In patients with dcSSc, reflux and bloating were the most prevalent symptoms (9/11, 81.8%), followed by diarrhea (5/11, 45.5%), constipation (5/11, 45.5%), and fecal soilage (2/11, 18.2%). There was no statistically significant difference in the prevalence of individual GI symptoms between patients with lcSSc and dcSSc, except for diarrhea, which was significantly more prevalent in dcSSc (lcSSc/dcSSc = 16.1%/45.5%, *p* = 0.04) (Table 2).

**Table 2:** Prevalence of gastrointestinal symptoms by disease subtype and autoantibody status in systemic sclerosis, assessed with the UCLA SCTC GIT 2.0 questionnaire

Gastrointestinal symptom	lcSSc (n=31)	dcSSc (n=11)	*p* value	ACA (n=26)	ATA (n=16)	*p* value
**Reflux**	20 (64.5%)	9 (81.8%)	0.45*	15 (57.7%)	14 (87.5%)	0.08*
**Distention/bloating**	19 (61.3%)	9 (81.8%)	0.28*	16 (61.5%)	12 (75%)	0.50*
**Constipation**	14 (45.2%)	5 (45.5%)	0.99#	10 (38.5%)	9 (56.3%)	0.26#
**Diarrhea**	5 (16.1%)	5 (45.5%)	0.04#	6 (23.1%)	4 (25%)	1.00*
**Fecal soilage**	2 (6.4%)	2 (18.2%)	0.27*	1 (3.8%)	3 (18.8%)	0.27*

*Note*. Abbreviations in use: lcSSc – limited cutaneous systemic sclerosis, dcSSc – diffuse cutaneous systemic sclerosis, ACA – anticentromere, ATA – anti-topoisomerase-I; *Fisher’s exact test, #Chi-square test.

The median score for diarrhea was significantly higher in patients with dcSSc compared to lcSSc (0.25 vs. 0.00, *p* = 0.02). Median scores for other GI symptoms did not differ significantly between the two disease subtypes. Although the median total GI score was higher in patients with dcSSc (0.45 vs. 0.15), the difference did not reach statistical significance (*p* = 0.05) (Table 3).

In ACA-positive patients, the most prevalent symptom was bloating (16/26, 61.5%), followed by reflux (15/26, 57.7%), constipation (10/26, 38.5%), diarrhea (6/26, 23.1%), and fecal soilage (1/26, 3.8%). In ATA-positive patients, reflux was the most prevalent symptom (14/16, 87.5%), followed by bloating (12/16, 75%), constipation (9/16, 56.3%), diarrhea (4/16, 25%), and fecal soilage (3/16, 18.8%). There were no statistically significant differences in the prevalence of individual GI symptoms according to the autoantibody status (Table 2). Similarly, the median UCLA SCTC GIT 2.0 subscale scores for reflux, bloating, constipation, diarrhea, and fecal soilage did not differ significantly between ACA-positive and ATA-positive patients. A trend toward higher total GIT scores was observed in ATA-positive patients compared to ACA-positive patients, although this difference was not statistically significant (Table 3).

**Table 3:** Median UCLA SCTC GIT 2.0 scores in systemic sclerosis patients by disease subtype and autoantibody status

UCLA SCTC GIT 2.0 scale	lcSSc (n=31) median (Q1-Q3)	dcSSc (n=11) median (Q1-Q3)	*P* value	ACA (n=26) median (Q1-Q3)	ATA (n=16) median (Q1-Q3)	*p* value
**Reflux**	0.35 (0.00-0.87)	0.50 (0.30-1.00)	0.34^†^	0.12 (0.00-0.84)	0.50 (0.32-0.91)	0.22^†^
**Distention/bloating**	0.50 (0.00-1.25)	0.50 (0.25-0.75)	0.94^†^	0.37 (0.00-1.25)	0.50 (0.18-0.87)	0.78^†^
**Constipation**	0.00 (0.00-0.50)	0.25 (0.00-0.37)	0.94^†^	0.00 (0.00-0.50)	0.25 (0.00-0.50)	0.62^†^
**Diarrhea**	0.00 (0.00-0.00)	0.25 (0.00-1.00)	0.02^†^	0.00 (0.00-0.00)	0.00 (0.00-0.25)	0.72^†^
**Fecal soilage**	0.00 (0.00-0.00)	0.00 (0.00-0.00)	0.27^†^	0.00 (0.00-0.00)	0.00 (0.00-0.00)	0.13^†^
**EWB**	0.00 (0.00-0.33)	0.27 (0.03-0.44)	0.47^†^;	0.00 (0.00-0.44)	0.22 (0.00-0.38)	0.71^†^;
**SF**	0.00 (0.00-0.41)	0.25 (0.16-0.45)	0.17^†^;	0.00 (0.00-0.46)	0.16 (0.00-0.41)	0.22^†^;
**Total UCLA SCTC** **GIT 2.0 score**	0.15 (0.05-0.41)	0.45 (0.22-0.53)	0.05^†^	0.13 (0.05-0.44)	0.40 (0.20-0.53)	0.15^†^

*Note*. Abbreviations in use: lcSSc – limited cutaneous systemic sclerosis, dcSSc – diffuse cutaneous systemic sclerosis, ACA – anticentromere, ATA – anti-topoisomerase-I; EWB – emotional well-being, SF – social functioning; † Mann–Whitney *U* test.

No statistically significant differences were observed in the impact of GI symptoms on social functioning or emotional well-being between the patients with lcSSc and dcSSc, or between ACA- and ATA-positive patients. The median values for social functioning and emotional well-being are shown in Table 3.

## Discussion

In this study, we assessed the prevalence and severity of GI symptoms in patients with systemic sclerosis and compared them according to the disease subtype and antibody profile. We also evaluated their impact on the social functioning and emotional well-being by using the validated Serbian version of the UCLA SCTC GIT 2.0 questionnaire [13]. We observed a higher prevalence of gastroesophageal reflux symptoms in patients with dcSSc compared to those with lcSSc (81.8% vs. 64.5%). However, this difference did not reach statistical significance. In previous studies using contrast radiography, objective signs of delayed esophageal transit were found significantly more frequently in patients with dcSSc compared to those with lcSSc (dcSSc/lcSSc = 85.5%/64%) [15]. In the EUSTAR cohort study, among more than 5,000 patients with SSc and interstitial lung disease (SSc-ILD), as many as 80.6% presented with gastroesophageal reflux disease (GERD) symptoms (reflux and/or dysphagia) at baseline [16]. Progressive smooth muscle atrophy and fibrosis of the esophageal wall lead to a delayed transit and gastroesophageal reflux. Similar pathological changes occur throughout the GI tract, contributing to bloating (stomach involvement), diarrhea (small intestine), constipation (colon), and fecal incontinence (anal sphincter). Recent histopathological studies confirm these findings in SSc [17]. In another EUSTAR cohort, older patients with diffuse disease developed GI symptoms earlier and more frequently [3].

In our study, bloating was observed in 61.3% of patients with lcSSc and 81.8% of those with dcSSc, without a significant difference in prevalence or severity, despite the literature data indicating that delayed gastric emptying is more commonly associated with the diffuse form of SSc [8]. In a 2022-dated Johns Hopkins cohort of 97 SSc patients, whole-gut scintigraphy demonstrated gastroparesis in 35% of cases, with delayed liquid gastric emptying showing a significant correlation with bloating and reflux symptoms on the UCLA SCTC GIT 2.0 [18]. The symptom indices for reflux and bloating had the highest median values among our patients, indicating that these were the most prevalent complaints (reflux, lcSSc/dcSSc = 0.35 vs. 0.50; bloating, lcSSc/dcSSc = 0.50 vs. 0.50). Zekovic and colleagues also reported the highest symptom index values for reflux and bloating, with a higher mean bloating index in ACA-positive patients compared to ATA-positive patients [13]. In contrast, we found no difference according to autoantibody status, which is consistent with previous studies showing no significant difference in bowel involvement between ACA- and ATA-positive patients [3,19].

In our study, diarrhea was significantly more prevalent and severe in patients with dcSSc compared to those with lcSSc, with a prevalence of 45.5% vs. 16.1% and the median diarrhea subscale scores of 0.25 vs. 0.00 (Tables 2 and 3).

One study involving 87 SSc patients found that 51% patients reported diarrhea, however, no difference in diarrhea prevalence was found between the lcSSc and dcSSc subtypes [20]. Diarrhea secondary to SIBO is common in SSc, and a recent systematic review and meta-analysis reported a pooled SIBO prevalence of approximately 40% in patients with SSc, with notable variation between studies depending on the diagnostic method [21]. Delayed intestinal transit contributes to the development of both SIBO and chronic intestinal pseudo-obstruction (CIPO) [22]. In a study involving 89 patients, SIBO was found to be associated with a disease duration of more than 5 years [23]. It is a cause of malabsorption and presents with diarrhea, bloating, weight loss, steatorrhea, and nutritional deterioration with deficiencies of iron, vitamin B12, and fat-soluble vitamins [24].

We found constipation in 45.2% of patients with lcSSc and in 45.5% of those with dcSSc, with no significant difference in symptom severity between the groups. Our findings are in line with those of Cano-Garcia et al., who reported a slightly higher prevalence of constipation, affecting 57.4% of their study population [25].

Fecal soilage results from fecal incontinence, which in SSc arises from smooth muscle atrophy and fibrosis of the internal anal sphincter [26]. Reported prevalence of this symptom in the literature varies widely, typically between 20% and 38%. For example, a French cohort study found fecal incontinence in 38% of SSc patients, while a Canadian study reported a prevalence of 27.2%, with 14.8% experiencing moderate to severe symptoms [26,27]. These figures may be influenced by selection bias, as studies often include patients from specialized centers, and the true prevalence may be underestimated due to patients’ reluctance to report sensitive symptoms [28]. In our study, symptoms of fecal soilage were reported by 6.4% of patients with lcSSc and 18.2% of those with dcSSc, and by 3.8% of ACA-positive patients and 18.8% of ATA-positive patients. Although fecal soilage symptoms were more prevalent in dcSSc patients, this difference did not reach statistical significance.

The presence and severity of GI disorders adversely affect patients’ social functioning, emotional well-being, and the quality of life. In our cohort, no significant differences were observed in the impact of GI symptoms on social functioning or emotional well-being between lcSSc and dcSSc subtypes, nor between ACA- and ATA-positive patients.

Our study has several limitations. It was conducted at a single center and included a relatively small number of patients, which may limit the generalizability of the findings and reduce the power to detect certain associations. Moreover, the cross-sectional design precludes any conclusions regarding causality. Although GI symptoms were assessed using a validated patient-reported outcome measure (UCLA SCTC GIT 2.0), this tool is based on subjective symptom perception and does not account for the objective severity or extent of GI involvement. As shown in previous studies, patients may have significant GI dysmotility even without symptoms, and the reported symptoms do not always reflect the underlying pathophysiology [29,30]. This may lead to an underestimation or overestimation of the true burden of GI disease in systemic sclerosis. Nevertheless, our study highlights the substantial impact of GI symptoms on patients’ quality of life and underscores the need for further investigation.

## Conclusions

Patients with diffuse cutaneous systemic sclerosis exhibited significantly higher prevalence and severity of diarrhea compared to those with limited cutaneous systemic sclerosis. In contrast, the prevalence and severity of other gastrointestinal symptoms did not differ significantly between the two disease subtypes. Similarly, no significant differences were observed in gastrointestinal symptom prevalence or severity between anticentromere-positive and anti-topoisomerase I-positive patients. Overall, gastrointestinal symptoms had a comparable impact on the social functioning and emotional well-being across the disease subtypes and antibody profiles.
